# Reorganization of the brain and heart rhythm during autogenic meditation

**DOI:** 10.3389/fnint.2013.00109

**Published:** 2014-01-13

**Authors:** Dae-Keun Kim, Jyoo-Hi Rhee, Seung Wan Kang

**Affiliations:** ^1^Data Center for Korean EEG, Seoul National UniversitySeoul, Korea; ^2^College of Nursing, Seoul National UniversitySeoul, Korea; ^3^J. H. Rhee Relaxation InstituteSeoul, Korea

**Keywords:** autogenic meditation, EEG, heart, coherence, relaxation, cortical reorganization

## Abstract

The underlying changes in heart coherence that are associated with reported EEG changes in response to meditation have been explored. We measured EEG and heart rate variability (HRV) before and during autogenic meditation. Fourteen subjects participated in the study. Heart coherence scores were significantly increased during meditation compared to the baseline. We found near significant decrease in high beta absolute power, increase in alpha relative power and significant increases in lower (alpha) and higher (above beta) band coherence during 3~min epochs of heart coherent meditation compared to 3~min epochs of heart non-coherence at baseline. The coherence and relative power increase in alpha band and absolute power decrease in high beta band could reflect relaxation state during the heart coherent meditation. The coherence increase in the higher (above beta) band could reflect cortico-cortical local integration and thereby affect cognitive reorganization, simultaneously with relaxation. Further research is still needed for a confirmation of heart coherence as a simple window for the meditative state.

## INTRODUCTION

Integrative neurophysiological substrate for transforming human attention and emotion system during meditation process are beginning to be understood by combining both central and peripheral system, brain, and body (Braboszcz et al., 2010; Brankovic, 2013; Kim et al., 2013). Self-regulation of attention is major component of meditation in which practitioners focus his/her attention on a particular physical/mental object in focused attention (FA) meditation and practitioners are instructed to allow any thought, feeling, or sensation to arise in consciousness while maintaining a nonreactive awareness to what is being experienced in open monitoring (OM) meditation (Lutz et al., 2008; Braboszcz et al., 2010). Slower respiration rate during meditation practice induces changes in cardiovascular activity that correspond to an increase in the activity of the restorative parasympathetic system (Takahashi et al., 2005; Wu and Lo, 2008a; Libby et al., 2012) or increased synchronization, or respiratory sinus arrhythmia (RSA), between the breathing cycle and the heartbeat during meditation (Cysarz and Büssing, 2005; Ditto et al., 2006b; Phongsuphap et al., 2008; Wu and Lo, 2010). Exercising cognitive control involves changing expectations or judgments about emotional stimuli. In FA practices, attention is focused away from the emotional reaction or emotion is simply being observed in OM practices. In addition, the RSA or parasympathetic activity could enhance emotion regulation by bottom-up process.

The diverse effects of meditation on the body and cognitive and affective processes have been reported so for long. Regular brief mindfulness meditation practice improved electrophysiological markers of allocating efficiency and self regulation of attention (Moore et al., 2012) and depth of information processing of trained meditators was more larger than non-meditators (van Leeuwen et al., 2012). Many studies revealed that meditation training not only increases brain efficiency in an attention task, but also improve emotion regulation through an attention training (Wadlinger and Isaacowitz, 2010; Kozasa et al., 2012). Furthermore, immune cell telomerase activity or mucosal immunity was reported to be modulated by intensive meditation training in a dose dependent fashion (Fan et al., 2010; Jacobs et al., 2011).

The meditation techniques are now used in clinical settings for patients suffering from emotional and attentional disorders. The practices have been adapted for clinical use based on traditional forms of meditation. Mindfulness-based stress reduction (MBSR) program is recommended as a useful method for improving mental health and reducing symptoms of stress, anxiety, and depression and also recommended in medical disease management to improve health-related quality of life. Mindfulness-based cognitive therapy (MBCT) is recommended for recovered recurrently depressed patients to prevent depressive relapse (Kabat-Zinn et al., 1992; Fjorback et al., 2011). HRV during meditation-based emotion exposure (MBEE) session combined with high levels of RSA achieved during meditation, turned out to be an excellent index of emotion regulation capacity, which is the capacity to feel, label, and accept emotions (Fabes and Eisenberg, 1997; Hallings-Pott et al., 2005). Recently, mindfulness meditation-related pain relief evidence for unique brain mechanisms in the regulation of pain was also reported (Zeidan et al., 2012).

The EEG signatures of meditation tend to be fairly complex across all bands and differ, as well, with the degree of the subject’s proficiency (Cahn and Polich, 2006). Expanding the current taxonomy of meditation and defining the characteristic neurophysiological signatures of various meditation categories are still important issues in meditation research (Josipovic, 2010; Travis and Shear, 2010) The central observation of this study is the basic course of Autogenic meditation, in other words, Autogenic training (AT). AT is a self-help relaxation technique easily practiced in daily life that consists of six standard exercises. The first exercise aims at muscular relaxation, which is achieved mainly by repeating a verbal formula to encourage heaviness. Subsequently, the concentration is focused passively on feeling warm, then calming the cardiac activity, slowed respiration, warmth in the abdominal region, and finally coolness in the head (Schultz and Luthe, 1969; Kanji et al., 2006). AT is a simple meditation practice, easily achievable in daily life, in which focused attention and OM are both incorporated. AT was not investigated so much in the EEG context, and there was only results for one or two channel EEG setting (Dierks et al., 1989; Jacobs and Lubar, 1989). Therefore, EEG signatures of Autogenic meditation including power and functional connectivity, especially coherence, across all frequency band from delta to gamma throughout the brain was thoroughly explored in this study. EEG signatures in specific regions of interest, are certainly important areas for further study.

The effect of Autogenic meditation on autonomic system has also been studied a little. High trait anxiety is associated with reduced HRV and vagal tone. In comparison to mental stress, AT increased HRV and facilitated the vagal control of the heart (Miu et al., 2009). AT significantly decreased cardiac sympathetic nervous activity and significantly increased cardiac parasympathetic nervous activity (Mitani et al., 2006). However, it remains controversial whether spectral analysis of HRV is really an appropriate index for autonomic nervous activity.

Most EEG changes observed during meditation have been increased power and synchronization of alpha, theta, and gamma band activities (Travis and Shear, 2010). However, the conventional HRV index has not shown consistent findings for meditative states (Ditto et al., 2006a; Conrad et al., 2007; Phongsuphap et al., 2008; Wu and Lo, 2008b). It might be due to increases in RSA during slow breathing phase of meditation contributing to the sympathetic power with a range of 0.04–0.15 Hz rather than parasympathetic power of 0.15–0.4 Hz, both bandwidth defined by Task force (Task-Force, 1996; Eckberg, 1997; Heathers, 2012; Kim et al., 2013).

There is another piece of evidence for the peripheral physiological activity that positive emotion is reflected by the novel index of the heart rate variability (HRV), especially, heart coherence (McCraty et al., 2009). The coherent pattern of heart rhythm, a sine wave-like heart rhythm oscillating at a frequency around 0.1 Hz, can be generated by sustained and self induced positive emotion, such as love, appreciation, and compassion (Bradley et al., 2010). It also facilitate higher cognitive functions. Therefore, heart coherence is expected to reflect a positive state of emotion as well as increases in RSA during the meditation.

Meditation training has been studied to understand underlying mechanism of homeostatic regulation based on independent consideration of EEG or HRV activity but has less on dependent consideration of both. Whereas the heart coherence and its dynamic correlation^1^ with EEG alpha band activity was investigated in the previous study (Kim et al., 2013), we focused on Autogenic meditation induced heart coherence changes and accompanied brain activity changes, especially, electroencephalography (EEG) power and coherence in the entire bandwidth from delta to gamma band. We tried to understand the mechanisms by which both the brain and peripherals are interacting in terms of the combination of both the brain and peripheral measures and also tried to expand current taxonomy of meditation and define the neurophysiological signatures of Autogenic meditation. Thereby it would be helpful to determine which meditation styles are more appropriate or helpful to particular person, finally.

We were particularly interested in whether the level of heart coherence as a simple biofeedback training modality could be a candidate surrogate marker for predicting high quality meditative states. This type of data could be useful as research moves forward for practical questions such as whether a simple ubiquitous sensor facilitates powerful meditation as a biofeedback modality and thus, help people recover from an extreme state of homeostatic depletion.

## MATERIALS AND METHODS

### PARTICIPANTS

A total of 14 autogenic meditators (*F* = 8, *M* = 6) were assessed (Mean ± SD = 43.5 ± 7.9 years, range = 29–59 years). These individuals had completed the 8 weeks autogenic standard training course, and all had been meditating daily at least for 1 month (Mean ± SD = 0.8 ± 0.5 years, range = 0.1–2.0 years) after they had finished their course. Participants were recruited from a local autogenic meditation community through word of mouth and email. The informed consent was obtained from each participant. The participants had no special cardiovascular or neurological conditions.

### RECORDING CONDITIONS

Electroencephalography data were collected by Brainmaster Discovery 24E Digital EEG system with a 19-channel ECI electrode cap from the following locations : Fp1, Fp2, F3, Fz, F4, F7, F8, T3, C3, Cz, C4, T4, T5, P3, Pz, P4, T6, O1, and O2. These scalp locations were referenced to the linked ear lobes, with the ground at the AFz. Impedances were kept below 10 kΩ. The signals were recorded with a band pass of 0.43–80 Hz and a digitization rate of 256 Hz. To monitor real-time ANS (autonomic nervous system) activity simultaneously with EEG data, a photoplethysmographic (PPG) sensor was attached over the index finger of the left hand by means of a flexible Velcro strap.

### PROCEDURE

The participants were instructed to sit on cushions and rest for 5 min for baseline measurements and meditate within the autogenic meditation process over a flexible period of time depending on their subjective meditation confirmation (Mean ± SD = 8.2 ± 3.6 min, range = 3–15 min). The participants kept their eyes closed during baseline measurements for comparing the resting state with meditative states.

### EEG ANALYSIS

The EEG data from each of the recording were first visually inspected with transient muscle- and movement-related artifacts removed. EEG power spectra were then computed by fast-Fourier transform (FFT) with hanning window based on 1024 points corresponding to 4 s epochs with a resolution of 0.25 Hz. Frequency bands were defined as follows: delta (δ), 2.0–4.0 Hz; theta (θ), 4.0–8.0 Hz; alpha (α), 8.0–12.0 Hz; beta (β), 12.0–25.0 Hz; High beta, 25.0–30.0 Hz; gamma (γ), 30.0–40.0 Hz; High gamma, 40.0–50.0 Hz. The absolute power of the EEG data during the baseline or the meditation condition is average value for all EEG epochs within the condition for a given bandwidth at a given location. The relative power for a given bandwidth is calculated from the absolute power for the bandwidth divided by total power at a given location. The total power is the summation of power across all the bandwidth at a given location. EEG power was averaged for all 19-channel electrodes.

Electroencephalography coherence was computed for all 171 intrahemispheric and interhemispheric pair wise combinations of electrodes. Mean square estimate of coherence was defined as

$${\Gamma_{X}^{2}}_{Y}\left(f\right)=\frac{G_{XY}{\left(f\right)}^{2}}{G_{XX}\left(f\right)G_{YY}\left(f\right)}$$

where Gxy(f) is the cross-power spectral density and Gxx(f) and Gyy(f) are the respective autopower spectral densities. Coherence was averaged for all pairwise combinations of the 19 channels for each of the seven frequency bands (delta, theta, alpha, beta, high beta, gamma, and high gamma).

The EEG of one participant had to be discarded due to technical problems. Data analysis was finally done on the EEGs from a group of 13 participants. In the power spectrum of EEG data, there was three sharp environmental noise peaks at 15, 30, and 44 Hz for all participants. The peak was very sharp frequency characteristics over the EEG spectrum and the peak frequency was identical for all subjects. Thus, the absolute power, relative power, and mean square estimate of the coherence was chunked by 1 Hz-bin and rejected the frequency component contaminated by the environmental noise peaks, and then added up to the values for a given bandwidth. All calculations were performed by using Matlab (Mathworks^®^ Matlab^®^ 7.13.0) and EEGLAB Toolbox (Delorme and Makeig, 2004).

### HRV ANALYSIS

Heart rate variability time series can be derived from the PPG data by peak detection algorithm. Power spectral density was obtained from the FFTs of the HRV time series. As described by other investigators, the power spectrum was divided into three major frequency ranges [low frequency (LF), medium frequency (MF), and high frequency (HF); McCraty et al., 1995]. The integral of the power spectrum within each region was calculated. The LF region (0.01–0.08 Hz) was primarily considered as the measure of the sympathetic activity with a minor parasympathetic component. In contrast, the HF region (0.15–0.5 Hz) was almost exclusively due to the parasympathetic activity. The LF/HF ratio was used as a measure of the sympathovagal balance. The MF region (0.08–0.15 Hz) has been used as an indicator of activity in the baroreceptor feedback loop controlling blood pressure. It is said that a measure of the MF power relative to the LF and HF regions is highly responsive to changing emotional states (McCraty et al., 1995).

### HEART COHERENCE ANALYSIS

Heart coherence was proposed as a novel index and formulated as follows (McCraty et al., 2009).

Peak⁢power/(total⁢ power−peak⁢power)

Peak power is determined by calculating the integral in a window 0.03 Hz wide, centered on the highest peak in the 0.04–0.26 Hz range of the HRV power spectrum. The total power is determined by calculating the integral in a window of 0.0033–0.4 Hz wide. Heart coherence thus approximates the MF/(LF + HF) ratio, assuming power in the range of MF is peak power.

A new index named “accumulative coherence score (ACS)” needs to be introduced. The accumulative coherent score is a method to assess how long the meditator is in a high coherent state defined by heart coherence. When the meditator is in a medium coherence state for each 5 s epoch, one point is added to the accumulative score, and when in a high coherence state, two points are added, and when in a low coherence state, one point is subtracted again for each 5 s epoch. (Heartmath^®^, HeartMath LLC, Boulder Creek, California.) Therefore, the longer the meditator is in a coherent state and stable in this state, the more accumulative coherence points the meditator has, resulting in a larger value for the “accumulative coherent score.”

### STATISTICAL ANALYSIS

The Wilcoxon signed rank test was used to compare baseline values with the values from the meditations of all participants. The Wilcoxon signed rank test, also known as the Wilcoxon matched pairs test, is a non-parametric test used to test the median difference in paired data. This test is the non-parametric equivalent of the paired *t*-test. Wilcoxon *p* values were taken from the table of critical values for the Wilcoxon singed rank test. Analyses were done in SPSS (IBM^®^ SPSS^®^ statistics version 21) with a significance level of 0.05. All data are represented as the mean ± standard deviation (SD).

## RESULTS

### ACCUMULATIVE COHERENT SCORE

**FIGURE 1:**
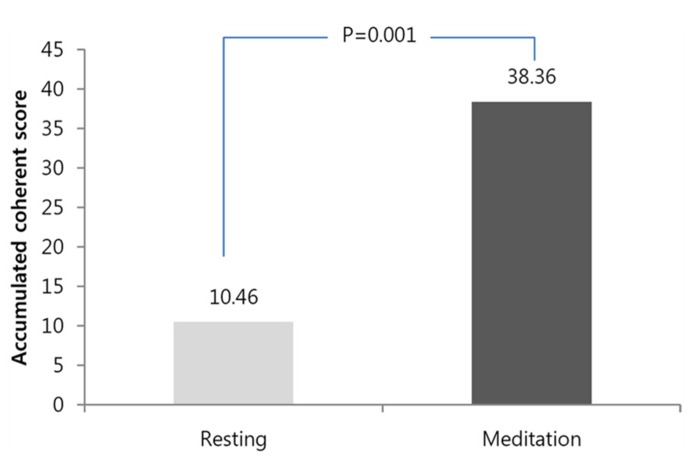
**Comparison of the accumulative coherence scores accumulative coherence scores (ACSs) between baseline and during meditation.** Significant increases were observed in the ACS. ACS means the longest length of an epoch in which each participant stayed in a heart coherent state.

The mean accumulative coherent score for the baseline measurement was 10.4 ± 8.1, and the mean accumulative coherent score for the meditation was 38.3 ± 25.1 (Wilcoxon test, *n* = 14, *Z* = 3.182, *p* = 0.001). **Figure 1** shows the statistical comparisons of the ACS for all participants between the baseline and during meditation. ACS is calculated for the maximum value the participant accomplished during each condition within the same time limit of 3 min.

A 3 min epoch from the baseline in which the ACS was not increasing and a 3 min epoch from the autogenic meditative state in which the ACS was increasing were selected. The two selected 3-min epochs were compared for subsequent statistical analyses of EEG power and coherence difference between the baseline and the meditative state. The epoch in which the ACS was not increasing indicates that the heart rhythm was not in a coherent ad stable state and vice versa.

### HRV INDEX

**FIGURE 2:**
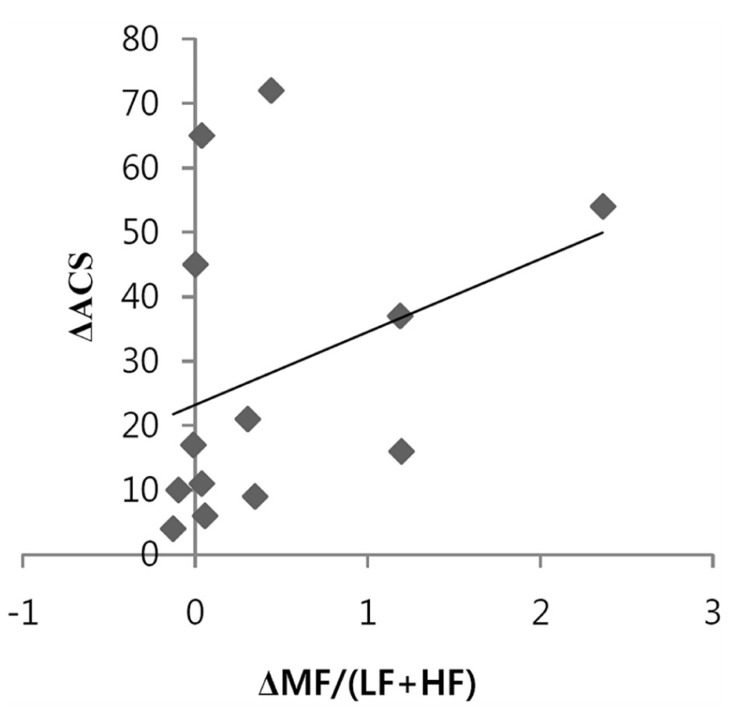
**Positive correlation between the changes of accumulative coherent scores (ACSs) and the changes in the MF/(LF + HF) ratios during 3 min epochs of heart coherent meditation compared to 3 min epochs of heart non-coherence at baseline (*n* = 13, *r* = 0.581, *p* = 0.037)**.

The relationship between the ACS and the conventional HRV index was explored. **Figure 2** shows a positive correlation between the increase in ACS and the increase in the MF/(LF + HF) ratio during meditation compared to the baseline was observed. This can be explained by the fact that heart coherence approximates the MF/(LF + HF) ratio. LF, MF, HF, and LF/HF did not significantly change during meditation compared to the baseline.

### EEG POWER

Significant decreases in the delta and near significant decreases in the high beta band absolute power during meditation compared to the baseline were observed. There were no significant changes in the other bands for absolute power including the theta, alpha, beta, gamma, and high gamma bands. In **Table 1**, the means and SD values for the EEG bands of absolute power for all the participants are presented for during 3-min epochs of heart coherent meditation and 3 min epochs of heart non-coherence at baseline with the final Wilcoxon analysis. In **Table 2** the means and SD values for the EEG bands of relative power for all the participants are presented for both condition.

**Table 1:** Mean and SD values of the EEG band absolute power for all participants for each condition with the final Wilcoxon analysis.

	Baseline	Meditation	Difference	*Z*	*p*-value
Delta	21.8 ± 10.2	16.3 ± 7.8	-5.5 ± 5.4	-1.99	0.046(*)
Theta	20.8 ± 9.7	18.7 ± 7.2	-2.0 ± 7.2	-1.01	0.311
Alpha	64.7 ± 43.6	64.7 ± 41.8	-0.1 ± 23.6	-0.31	0.753
Beta	19.1 ± 7.5	18.5 ± 8.6	-0.6 ± 4.6	-0.87	0.382
High beta	2.3 ± 1.1	2.0 ± 1.1	-0.3 ± 0.5	-1.99	0.055(*)
Gamma	3.2 ± 0.9	2.9 ± 1.3	-0.3 ± 0.9	-0.94	0.345
High gamma	2.2 ± 1.2	1.9 ± 1.2	-0.3 ± 0.9	-1.15	0.249

*There were significant decreases in the delta and near significant decrease in the high beta band absolute power during 3 min epochs of heart coherent meditations compared to 3 min epochs of heart non-coherence at baseline.
*

**Table 2:** Mean and SD values of the EEG band relative power for all participants for each condition with the final Wilcoxon analysis.

	Baseline	Meditation	Difference	*Z*	*p-*value
Delta	17.3 ± 7.0	14.1 ± 6.5	-3.1 ± 3.5	-2.62	0.009(**)
Theta	16.1 ± 4.8	15.9 ± 5.4	-0.2 ± 3.1	-0.03	0.972
Alpha	44.8 ± 14.9	48.0 ± 15.8	3.1 ± 6.0	1.5	0.133
Beta	15.2 ± 5.4	15.9 ± 8.1	0.7 ± 4.0	0.03	0.972
High beta	1.8 ± 0.7	1.7 ± 0.8	-0.1 ± 0.6	-0.94	0.345
Gamma	2.7 ± 1.0	2.4 ± 1.0	-0.2 ± 1.2	-0.03	0.972
High gamma	1.8 ± 1.1	1.6 ± 1.0	-0.1 ± 1.1	-0.31	0.753

*There were significant decreases in the delta and increase in the alpha band relative power during 3 min epochs of heart coherent meditations compared to 3 min epochs of heart non-coherence at baseline.
*

### EEG COHERENCE

Significant increases in the alpha, beta, high beta, gamma, and the high gamma band coherence averaged over 171 channel combinations during meditation compared to the baseline were observed. There were no significant coherence changes in the other bands including the delta, theta bands. In **Table 3**, the mean and SD values of the EEG band coherence for all participants are presented for during 3-min epochs of heart coherent meditation and 3 min epochs of heart non-coherence at baseline with the final Wilcoxon analysis.

**Table 3:** Mean and SD values of EEG band coherence for all participants are presented for each condition with the final Wilcoxon analysis.

	Baseline	Meditation	Difference	*Z*	*p*-value
Delta	42.6 ± 11.9	41.4 ± 8.2	-1.2 ± 7.1	-0.594	0.552
Theta	41.6 ± 6.8	42.5 ± 7.4	0.9 ± 4.2	0.454	0.65
Alpha	49.4 ± 5.6	51.5 ± 6.6	2.1 ± 3.7	1.992	0.046 (*)
Beta	35.7 ± 4.6	37.0 ± 5.3	1.3 ± 2.1	1.992	0.046 (*)
High beta	32.5 ± 4.5	34.4 ± 5.1	1.9 ± 2.8	1.992	0.046 (*)
gamma	31.7 ± 5.1	34.5 ± 7.0	2.9 ± 3.6	2.621	0.009 (**)
High gamma	37.0 ± 7.2	39.6 ± 7.6	2.6 ± 3.9	1.992	0.046 (*)

*There were significant increases in the lower frequency band (alpha) coherence and higher frequency band (above beta) coherence during 3 min epochs of heart coherent meditation compared to the 3 min epochs of heart non-coherence at baseline.*

## DISCUSSIONS

We found that the ACS was significantly increased during autogenic meditation compared to the baseline. In addition, significant decrease in the delta bands and near significant decrease in high beta bands for absolute power, significant decrease in delta bands and increases alpha bands for relative power and significant increases in the alpha, beta, high beta, gamma, and high gamma bands for coherence were observed during 3 min epochs of heart coherent meditation compared to 3 min epochs of heart non-coherence at baseline (**Tables 1–3**).

There has been a close positive relationship between EEG power in beta band and metabolic PET perfusion activity in the corresponding cortical area of the human brain (Cook et al., 1988; Singh et al., 2002). This relation was reversed for the alpha band which showing alpha frequencies are idling rhythms of sensory systems and synchronization at 10 Hz frequency indicates the state of the sensory system when neurons do not relay sensory information but ready to commence when a relevant stimulus will appear. Binding role of gamma oscillation for combining different features of an object into a single percept was experimentally demonstrated in animal experiment (Gray et al., 1989; Gray and Singer, 1989). Recently, it was speculated that neuronal synchrony may be also critical for conscious processing (Engel and Singer, 2001). In humans, scalp and intracranial EEG recordings consistently reveal the existence of synchronized oscillatory activity in the gamma range when subjects experience a coherent visual percept (Tallon-Baudry and Bertrand, 1999; Tallon-Baudry et al., 2005).

Coherence is a statistical measure of phase consistency between two time series. This indicates that the network properties of shared information and coupling as reflected by EEG coherence (Thatcher et al., 2005). High coherence between two EEG signals has been interpreted as reflecting a strong structural or functional connection between the underlying cortical regions (Barry et al., 2011). It can be assumed that coherence in different frequency bands reflects different neuronal networks and neuronal processing, which can be involved in cortico-cortical and thalamo-cortical circuits (Wang, 2010). An attempt to relate functional aspects of neural integration to different frequencies proposed that higher frequencies are involved in short-range integration, while lower frequencies are involved with longer-range integration (von Stein and Sarnthein, 2000). Increasing trend of beta (13–25 Hz) coherence in the developmental EEG database was also revealed which showing higher frequency coherence represent increased local integration during maturation from child to adolescent (Thatcher et al., 2008).

The neuronal mechanism of lower frequency rhythm generation is generally originated from thalamo-cortical circuits. Delta oscillations are associated with a so-called burst mode of thalamo-cortical cells. Theta rhythm are generated in septo-hippocampal network with thalamo-cortical circuits. The cortex receives inputs from the ventral posterior nucleus of the thalamus generate alpha oscillations in the sensory-motor cortex and the occipital and parietal areas receive inputs from the LGB and pulvinar generate occipital and parietal alpha, respectively (Juri, 2009). Whereas, the neuronal mechanism of higher frequency rhythm generation is due to local inhibitory feedback neurons located inside of the neocortex and the rhythm oscillate between the different layers of the neocortex up to about 300 Hz.

The word “gamma” is not specific to a particular frequency band and is used by many people to describe different frequency ranges and the neuronal mechanism is still controversial. Nunez and Lopes da Silva consider 25 Hz and higher as gamma and others consider 30 Hz and higher and others 40 Hz and higher, etc. The suggested mechanism for gamma waves is that the waves originating in the thalamus, sweeps the brain from front to back, 40 times per second, drawing different neuronal circuits into synch with the precept, and thereby bringing the precept into the attentional foreground (Pollack, 1999). The synchronization of neuronal discharges can serve for the integration of distributed neurons into cell assemblies and that this process may underlie the selection of perceptually and behaviorally relevant information (Engel et al., 1999). About 40 Hz rhythms have been measured in the reticular ventral-tegmentum and in other brainstem regions (Montaron et al., 1982) indicating that local cortical circuits modulated by the reticular formation and cortico-cortical connections are responsible for localized gamma activity. Although Gray et al. (1989) failed to find 40 Hz oscillations in the thalamus which is also what was reported by Lopes da Silva et al. (1970), synchronization of fast (30–40 Hz) spontaneous oscillations in intrathalamic and thalamo-cortical networks was reported (Steriade et al., 1996). Coherence of gamma-band EEG activity as a basis for learning was proposed (Miltner et al., 1999; Yordanova et al., 2002) and relations to the human cognitive processing has been investigated (Kaiser and Lutzenberger, 2003, 2005). It was also suggested that 40 Hz is especially important and is thought to be associative peak performance (Thompson and Thompson, 2003). This was also manifested by some meditation study in which meditation masters have the ability to put the brain into a state in which it is a maximally sensitive (Lutz et al., 2004). Therefore, we could estimate that increase of gamma band activity can be associated with reorganization of cortical oscillations or learning during the meditation practice. In animal study, it was shown that gamma oscillations are generated by synchronous activity of fast-spiking inhibitory interneurons, can be induced intentionally *in vivo* by cell-type-specific activation (Steriade et al., 1990; Cardin et al., 2009). However, more fundamental process beyond cortical oscillations are quite limited so far in human study. Although functional neuroimaging can show metabolic changes in specific regions, which is not a direct reflections of neuronal activities and its time resolution is too low.

Regarding high frequency coherence representing local short range integration in the cortical circuits, the higher coherence in beta and above frequency range during the heart coherent meditation could reflect a stronger short range structural or functional connection between the underlying cortical regions. Regarding lower frequency coherence representing long range integration such as thalamo-cortical circuits (Sarnthein and Jeanmonod, 2008), the higher coherence in alpha frequency range during the heart coherent meditation could reflect a stronger long range structural or functional connection between the underlying thalamo-cortical regions.

Although it is not yet possible to assign a specific functional role to each frequency, the presence of beta and gamma oscillations is thought to represent an activated state of the underlying neuronal network. As in this study, some researchers proposed the possibility that beta and gamma oscillations accompany alpha increases (Lansbergen et al., 2011). The EEG as well as its functions and interdependencies between frequency band throughout the brain and in specific regions of interest, are also certainly important areas for further study.

Regarding high beta power decrease in the heart coherent meditation, it was reported so for long high beta power had been associated with stress and anxiety (Thompson and Thompson, 2003, 2007). There was also a recent study where significant positive correlation between the salivary cortisol level and high beta power and a significant negative correlation between SDNN and relative high beta power during an eyes-closed resting condition (Seo and Lee, 2010). This result was also in line with the findings that sufferers of chronic stress have typically having lower HRV and higher cortisol levels.

The delta frequency is most notably associated with the onset of sleep (Lubin et al., 1973). However, it is also suggested that delta activity plays a particular role in information encoding and retrieval as well as in overall intelligence. Bursts in both delta (2 Hz) and gamma (100 Hz) bands resulted in maximal long term-potentiation in the neocortex (Teyler, 1989). Activity of neocortical neurons during slow-wave sleep is associated with neuronal plasticity and may playa role in consolidating memory traces acquired during the waking state (Steriade, 2004). Klimesch et al. (2006) identified a role of delta frequency activity in a two stage episodic encoding process occurring between repeated learning trials. Traces are first processed at parietal sites at approximately 300 ms. Then, further processing takes place in regions of the medial temporal lobe at approximately 500 ms. Only the first stage is associated with theta, whereas the second is characterized by a slow wave with a frequency of approximately 2.5 Hz (Klimesch et al., 2006). It might be considered that delta plays an important part in the memory consolidation function due to its prominence in the limbic system (Smythies, 1966) and in arousal due to the associated connections from the reticular formation. The delta frequency has not been thought of as important to cognitive processes, however, as the mysteries associated with this frequency domain are unraveled, it may be that delta plays an important role in cognitive functioning as well as emotion and regulatory processes with cross-frequency co-modulation properties (Cannon, 2012).

Our results show that the lower frequency band (alpha) and higher frequency band (above beta) coherence significantly increased during meditation compared to the baseline. These results could represent the meditation facilitate increasing longer range integration like thalamo-cortical integration and cortico-cortical local integration simultaneously. The increased local range integration, more evident in the gamma band, may be related to the cortical-cortical process of functional reorganization, and thereby modulating cognitive domain. The increased alpha coherence may be related to the process of relaxation. The findings of increased alpha relative power and decreased high beta absolute power also indicate the relaxation effect. Taken together, our findings on autogenic meditation associated EEG changes could provide evidence for the neural basis of meditation facilitating a specific state of consciousness state in which deep relaxation and increased cortical reorganization coexist. The significant delta power decrease will be more evident when cognitive functioning of delta band as well as emotion and regulatory processes with cross-frequency co-modulation properties are more unraveled.

More dynamic aspects of this heart brain connection, windowing every 4 s during both baseline and meditation rather than 3 min herein, between heart coherence and EEG activities, especially for alpha band were thoroughly investigated elsewhere (Kim et al., 2013). Some investigators have asserted that MF/(LF + HF) ratio highly and sensitively represents emotional status (McCraty et al., 1995) rather than the conventional HRV index (Task-Force, 1996). Heart coherence, approximating the MF/(LF + HF) ratio, might predict stable and high quality meditative states and thereby be a novel meditation related physiology marker.

According to polyvagal theory, vagal afferents from the heart feed back to the nucleus of the solitary tract in the medulla which is connected to the parabrachial nucleus and the locus coeruleus. These nuclei connect to the forebrain with links to the hypothalamus, amygdala, thalamic connections to the insula, orbitofrontal, and prefrontal areas, all of which give feedback to the anterior cingulate (AC; Porges, 2003, 2007). Theoretically this could synergistically assist in normalizing the activity of the AC and its connections through both the mirror neuron system and the limbic system (Thompson and Thompson, 2007).

The heart coherent states in which the heart rate accelerates during inhalation and decelerate during exhalation, so called RSA, can be easily achieved by slow diaphragmatic breathing at about six breaths per minute (faster in children). Although neuro-scientific substrates for the emotion mostly lies very deep in the brain so it is very difficult to quantify this area, we could estimate that the heart coherent state could facilitate both relaxation and cortical reorganization through the functional anatomical pathway according to the result of this study.

A large part of science operates as if cognition and emotion are only regulated by the central nervous system (CNS). The psychological construction of emotion can be fully understood only by integrating complex causal chain linking central and peripheral psychophysiology (Russell, 2003). It was argued that this meditative state enhance synchronization of heart coherence and EEG alpha activity (Kim et al., 2013) but it is still not clear how and how much this reorganized heart and brain state enhance cognitive functioning and emotional wellness and hard to determine whether heart coherent state drive relaxed and cortical reorganization state or vice versa. Further research will be needed.

## Conflict of Interest Statement

The authors declare that the research was conducted in the absence of any commercial or financial relationships that could be construed as a potential conflict of interest.

## Footnotes

1 Heart coherence(~0.1Hz centered) is very slow varying signal compared to EEG signal(~10Hz centered). Therefore, dynamic correlations was monitored in which 60 s of HRV tachogram data were epoched successively with an overlap of 56 s and 4 s of EEG data were epoched successively without overlapping. In this study, average EEG signatures was investigated rather than dynamic pattern, from alpha to all frequency bands, during 3 min of heart coherent epoch at meditation compared to 3 min of heart non-coherent epoch at baseline.
